# Combined Cerebrospinal Fluid Neurofilament Light Chain Protein and Chitinase-3 Like-1 Levels in Defining Disease Course and Prognosis in Multiple Sclerosis

**DOI:** 10.3389/fneur.2019.01008

**Published:** 2019-09-23

**Authors:** Sara Gil-Perotin, Jessica Castillo-Villalba, Laura Cubas-Nuñez, Raquel Gasque, David Hervas, Josep Gomez-Mateu, Carmen Alcala, Francisco Perez-Miralles, Francisco Gascon, Jose Andres Dominguez, Bonaventura Casanova

**Affiliations:** ^1^Multiple Sclerosis Unit, Hospital Universitari i Politècnic La Fe, Valencia, Spain; ^2^Research Group in Neuroimmunology, Health Research Institute La Fe, Valencia, Spain; ^3^Biostatistics Unit, Health Research Institute La Fe, Valencia, Spain; ^4^Neurology Department, Hospital Universitari Dr. Peset, Valencia, Spain; ^5^Neuroimmunology Unit, Hospital Clínic de València, Valencia, Spain

**Keywords:** YKL-40, CHI3L1, NFL, progressive multiple sclerosis, gadolinium-enhancing lesions

## Abstract

**Background:** Neurofilament light chain protein (NFL) and chitinase3-like1 (CHI3L1) have gained importance recently as prognostic biomarkers in multiple sclerosis (MS).

**Objectives:** We aimed to investigate NFL and CHI3L1 cerebrospinal fluid (CSF) profiles in multiple sclerosis and the informative and prognostic potential of the individual and combined measures.

**Methods:** CSF NFL and CHI3L1 levels were measured in a cross-sectional cohort of 157 MS patients [99 relapsing-remitting (RRMS), 35 secondary progressive (SPMS), and 23 primary progressive (PPMS)]. Clinical relapse and/or gadolinium-enhanced lesions (GEL) in MRI within 90 days from CSF collection by lumbar puncture (LP) were registered and considered as indicators of disease activity. Longitudinal treatment and disability data were evaluated during medical visits with a median follow-up of 50 months.

**Results:** CSF levels of NFL and CHI3L1 were higher in MS patients compared to non-MS controls. In RRMS and SPMS patients, increased NFL levels were associated with clinical relapse, and gadolinium-enhanced lesions in MRI (*p* < 0.001), while high CHI3L1 levels were characteristic of progressive disease (*p* = 0.01). In RRMS patients, CSF NFL, and CHI3L1 levels correlated with each other (*r* = 0.58), and with IgM-oligoclonal bands (*p* = 0.02 and *p* = 0.004, respectively). In addition, CSF CHI3L1 concentration was a predictor for 1-point EDSS worsening {HR = 2.99 [95% CI (1.27, 7.07)]} and progression during follow-up {HR = 18 [95% CI (2.31, 141.3)]}. The pattern of combined measure of biomarkers was useful to discriminate MS phenotypes and to anticipate clinical progression: RRMS more frequently presented high NFL combined with low CHI3L1 levels, compared to SPMS (HR 0.41 [0.18–0.82]), and PPMS (HR 0.46 [0.19–0.87]), while elevation of both biomarkers preceded diagnosis of clinical progression in RRMS patients (log rank = 0.02).

**Conclusions:** Individual measures of CSF NFL and CHI3L1 are biomarkers of disease activity and progression, respectively. The pattern of combined measure discriminates MS phenotypes. It also predicts the subset of RRMS patients that will progress clinically allowing early intervention.

## Introduction

Neurofilament light protein (NFL), a cytoskeletal polypeptide of the axon (1), and chitinase 3-like 1 (CHI3L1- also known as YKL40 or gp39), a glycoprotein secreted by activated glia in the central nervous system (CNS) (2), have both shown to be biomarkers of axonal destruction, and inflammation in multiple sclerosis (MS), respectively.

NFL is not a specific biomarker but a reflection of axonal destruction in several neurological diseases (3–6). In MS, cerebrospinal fluid (CSF), and serum levels of NFL have been suggested as markers for disease activity in MS (7) and predictors of clinically isolated syndrome (CIS) conversion to MS (8–11). NFL is also considered as a prognostic marker of worse outcomes regarding brain atrophy (12, 13) and disability progression (13). Its levels in serum and CSF decrease with Disease-Modifying Therapies (DMT), therefore, it seems to be a good surrogate marker for measuring response to treatment (14–18).

CHI3L1 expression is not restricted to the CNS, but its presence in the CSF has been related to endogenous secretion by astrocytes, and microglia/monocytes (19). Its increase has been related to CIS conversion to MS (20, 21), advanced (20), and/or progressive disease (7, 22–24), cognitive impairment (25), and increased disability (20, 24). Like NFL, CHI3L1 levels were responsive to DMTs in relapsing MS (15, 26–28).

In relapsing-remitting MS (RRMS), the presence of CSF oligoclonal IgM bands (OCMB) has been associated with more substantial T2 lesion load, increased gadolinium-enhancing lesions (GEL), higher relapse rate, more neurological disability, and brain volume loss over time. OCMB have also been related to earlier conversion to secondary progressive MS (SPMS) and can help to identify a subset of primary progressive MS (PPMS) patients with a more inflammatory phenotype (29–31).

Although literature regarding the role of NFL and CHI3L1 in MS is profuse, there is limited information about their relationship with OCMB and little is known about the diagnostic and predictive role of the combined assessment of CSF levels of both NFL and CHI3L1. In this work, with a cohort of 157 patients including RRMS and progressive phenotypes (SPMS; PPMS) and with prospectively collected disability data, we aimed to demonstrate that the combined measure of both biomarkers in the CSF might have value, not only in identifying distinct MS phenotypes but also in predicting accrual of disability and further diagnosis of progressive disease in RRMS patients.

## Methods

### Study Cohort

**Figure 1:**
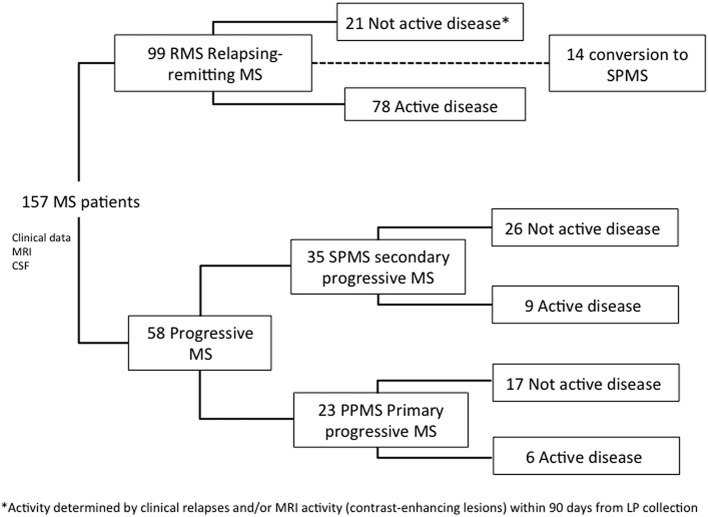
Flow chart of study cohort.

The study included all MS patients with available samples of CSF, magnetic resonance imaging (MRI), and longitudinal disability data, seen at two university hospitals in Valencia, Spain (Hospital Universitari I Politècnic La Fe and Hospital Clinic Universitari) between 2008 and 2017 (Figure 1). Demographic and clinical data were retrospectively collected with the last update in December 2018. All patients provided written informed consent. Non-MS Control CSF samples were selected from patients who were seen because of headache with fever, Pseudotumor cerebri, encephalopathy, or dementia [median age: 33 (IQR, 29–35); 59% female]. All patients in this cohort had a normal brain MRI and CSF analysis, with no evidence of infection, inflammation, autoimmunity, or known neurodegenerative disease. The study was approved by the Institutional Ethics Committee in Hospital Universitari I Politècnic La Fe (reference number PI17/01544).

### Definitions

Diagnosis of clinically definite MS was made according to 2017 McDonald criteria (32). Active disease was considered when a clinical attack occurred and/or at least one gadolinium-enhanced lesion (GEL) was present in T1-weighted MRI. A clinical attack or relapse was defined as an acute worsening of neurologic function lasting more than 24 h, not explained by fever or physical stress, and followed by a variable degree of recovery. Urinary symptoms alone were not considered for a diagnosis of relapse. CSF samples were considered contemporary to active disease when a lumbar puncture (LP) was performed within 90 days after assessment of clinical attack and/or GEL. Clinical phenotypes were classified according to modified Lublin criteria (33). A secondary progressive phase of multiple sclerosis (SPMS) was considered when patients with an EDSS score ≥3.0 had a 6-month confirmed increase to an EDSS score of ≥4.0, pyramidal functional system was ≥2.0, and there was no evidence of relapse. PPMS phenotype was assigned to those patients who fulfilled 2017 McDonald criteria for PPMS (34). Neurological disability was defined as the neurological worsening, derived not only from clinical relapse but also from accrual of neurological symptoms in the absence of relapse or radiological signs of acute inflammation. It was estimated according to the expanded disability status scale (EDSS) (35) at the time of LP and every 6 months until the last visit. One-point EDSS worsening was considered anytime during follow-up as a measure of neurological disability, secondary to acute inflammation, or to progression-related accrual of disability. Treatment failure was considered as a loss of NEDA-3 status (a composite measure of disease activity based on the absence of relapses, no evidence of disability progression by the EDSS, and no new T2 lesions or GEL in MRI) (36).

### Treatment

Patients with clinically definite MS were treated with first-line DMT, chosen at physician discretion, unless one of the following circumstances occurred: (i) two clinical attacks in 1 year, (ii) a clinical attack and/or a new GEL within 3 months after the bout, (iii) a disabling clinical attack with residual EDSS of at least 2 points. In these cases, and those with treatment failure, second line DMT was administered. Non-responders to first- and second line DMT proceeded to autologous stem cell transplantation (ASCT).

### CSF Sampling and Biomarker Analysis

CSF samples were stored at −80°C in the Biobank La Fe with the approval of the Ethics and Scientific Committees (PT17/0015/0043). NFL and CHI3L1 levels in CSF were assessed by enzyme-linked immunosorbent assays using commercially available kits according to manufacturer's instructions (Uman Diagnostics AB, Umea, Sweden and Quantikine ELISA kit, R&D Systems, respectively). The mean intra-assay coefficients of variation for NFL and CHI3L1 were 4.5 and 6.5%, and inter-assay 3.3 and 5.2%, respectively.

### Other Ancillary Tests

Brain and spinal cord 1.5/3.0 Tesla MRI were performed at diagnosis and during the follow-up. CSF oligoclonal IgG bands and oligoclonal IgM bands (OCMB) against lipids were studied by isoelectric focusing and immunoblotting, as previously described (29). Serum and CSF were tested to rule out infections, other inflammatory diseases, and non-MS demyelinating diseases.

### Statistical Analysis

Statistical analysis was performed by the Biostatistics Department of the Research Health Institute La Fe. Categorical variables were described by counts (n) and percentages (%), continuous, and ordinal variables by median (first and third quartiles). CSF NFL/CHI3L1 levels were log-transformed (logNFL and logCHI3L1) to meet the normality assumption. Comparisons between groups were performed with U-Mann Whitney, Student *t*-test, and a one-way analysis of covariance (ANCOVA) using age, sex, and disease duration as covariates, with no significant changes in the estimated effects found for NFL, and CHI3L1. Bonferroni *post-hoc* correction method was performed for multiple comparisons. Correlations were analyzed with the Pearson's product-moment correlation test. Spearman's rank correlation method was additionally performed as a sensitivity analysis. To analyze the contribution of paired values of biomarkers, patients were categorized into four groups with a median split method (the median value was calculated from the RRMS cohort without disease activity): group 1 included patients with CSF NFL and CHI3L1 levels below median values, group 2 and 3 included patients with only NFL or CHI3L1 values above median, respectively, and group 4 comprised patients with CSF NFL, and CHI3L1 concentrations above median levels. Bivariate analysis with Kaplan Meier survival curves was performed for the probability of 1-point EDSS worsening, diagnosis of progressive disease, escalation of treatment, and occurrence of first relapse after LP in RRMS patients. Predictors were age, sex, disease activity, EDSS, logNFL, and logCHI3L1 (or alternatively patient's biomarker categories), presence of OCMB in the CSF, and treatment at the time of LP. Elastic net penalized Cox proportional hazard regression models were performed in the RRMS subgroup for the same outcomes and predictors. After the selection of the predictors with the elastic net algorithm, the model was refitted with a standard Cox regression to get approximate *p*-values and estimates of the effects. Additionally, a multinomial logistic regression model was performed including log-transformed CSF NFL, and CHI3L1 levels as predictors. MS clinical phenotypes (RRMS, SPMS, and PPMS) and diagnosis of SPMS were used as response variables with RRMS as the reference group. A plot of marginal effects was drawn to ease the interpretation of multinomial logistic regression model. All analyses were conducted using SPSS 21.0 v and R version 3.4.3 (The CRAN project).

## Results

### Patient Characteristics

We studied 157 MS patients, 99 RRMS (63%), and 58 progressive MS (37%), of which 35 were SPMS (22%), and 23 PPMS (15%) (Figure 1).

Clinical and demographic features are shown in Table 1. Clinical phenotypes differed in age at disease onset and at time of LP, time lapse between disease onset and LP, gender, disease activity (relapse or/and GEL), and EDSS. The median follow-up time after LP was also significantly different between groups. In 28 patients, LP was performed within 90 days after disease onset.

**Table 1:** Demographics and CSF findings.

**Variable**	**RRMS (*n* = 99)**	**SPMS (*n* = 35)**	**PPMS (*n* = 23)**	***p***
Age at disease onset (years)	28 (23.5, 36.5)	29 (23, 36)	41 (34.5, 47.5)	<0.001
Age at time of LP (years)	35 (29.5, 41)	45 (38.5, 50)	51 (41, 56)	<0.001
Female gender	79 (79.8%)	21 (60%)	10 (43.5%)	<0.001
Time from diagnosis to LP (years)	2.1 (0.3, 9.8)	15.8 (8.8, 19.3)	8.3 (5.0, 11.8)	<0.001
Follow-up from LP (years)	4.4 (3.0, 5.9)	4.3 (2.3, 5.7)	3.1 (2.5, 5.7)	<0.001
Clinical relapse at time of LP	53 (53.5%)	2 (5.88%)	1 (5%)	<0.001
GEL in MRI at time of LP	53 (56.4%)	8 (25.81%)	6 (30%)	<0.001
OCB-IgG	91 (91.9%)	31 (88.6%)	22 (95.7%)	1
OCB-IgM	49 (49.5%)	18 (51.4%)	10 (43.5%)	0.9
Baseline EDSS	2 (1, 3)	5.5 (4.25, 6.5)	5 (3.5, 6)	<0.001
NFL in CSF (pg/ml)	591.9 (290, 1106)	533 (266, 678)	450 (304, 746)	0.6
CHI3L1 in CSF (ng/ml)	118.97 (81, 186)	139.55 (96, 212)	180.25 (146, 265)	0.01

*RRMS, relapsing-remitting multiple sclerosis; SPMS, secondary progressive multiple sclerosis; PPMS, primary progressive multiple sclerosis; LP, lumbar puncture; GEL, gadolinium- enhancing lesions; OCB, oligoclonal bands; NFL, neurofilament light chain; CHI3L1, chitinase-3-like-1; CSF, cerebrospinal fluid. Categorical variables are described by counts (n), and percentages (%), continuous and ordinal variables by median (first and third quartiles). U Mann Whitney test was used in all continuous variables except in NFL and CHI3L1 in which logNFL and logCHI3L1 were analyzed with T-student test. χ^2^ test was used for categorical variables*.

Ninety-three patients (60%) had documented clinical attack and/or GEL in the MRI within 90 days from LP. Median time from a clinical attack to LP and from MRI to CSF collection was 38 days (14, 65) and 22 days (5, 50), respectively. At the time of analysis, 14 patients in the RRMS cohort had sustained an increase in disability without clinical attacks and, therefore, were reassigned to SPMS phenotype. Disease duration in these patients was significantly higher compared to that in patients who did not progress during follow-up (*p* = 0.03).

### CSF Levels of NFL and CHI3L1 in MS and Relationship With Disease Inflammatory Activity or Disease Course

**Figure 2:**
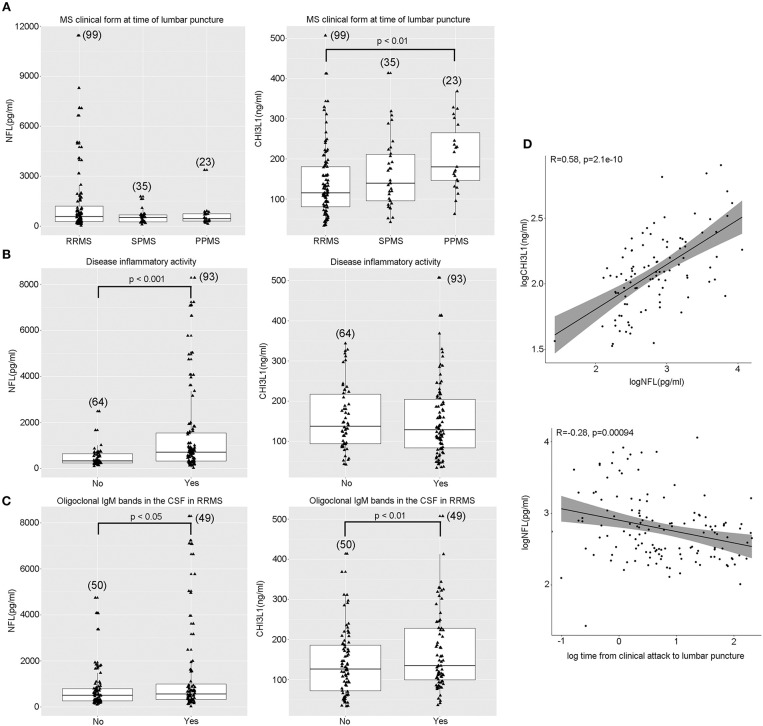
CSF levels of NFL and CHI3L1 in MS in association to phenotype, inflammatory activity, and other influencing variables. **(A)** Patients with disease activity had higher CSF NFL levels compared to patients without documented disease activity (defined as clinical attack and/or GEL in the MRI within 90 days of CSF collection) (Student *T*-test *p* < 0.001). CHI3L1 levels were not different with regards to disease activity (Student *T*-test *p* = 0.705). **(B)** NFL levels were not different between MS forms (ANOVA, *p* = 0.6) while CHI3L1 were increased in progressive MS compared to RRMS (ANOVA *p* = 0.01; Bonferroni *post-hoc* RRMS vs. PPMS *p* = 0.009). **(C)** Both NFL and CHI3L1 were higher in the presence of OCMB in the RRMS cohort (Student *T*-test *p* = 0.02 and *p* = 0.004, respectively). **(D)** NFL and CHI3L1 correlated between each other (Pearson's *r* = 0.58; *p* < 0.001) in the RRMS cohort. NFL levels were lower as time from clinical relapse passed (Pearson's *r* = 0.29; *p* = 0.04). Sample size for each condition is expressed between brackets in the diagram. Student *T*-test and correlations were performed with log-transformed NFL and CHI3L1. NFL, neurofilament light chain; CHI3L1, chitinase-3-like-1; RRMS, relapsing-remitting multiple sclerosis; SPMS, secondary progressive multiple sclerosis; PPMS, primary progressive multiple sclerosis. GEL, gadolinium-enhanced lesions; CSF, cerebrospinal fluid; MRI, magnetic resonance imaging.

Overall, the median CSF levels of NFL were 536 pg/ml (289, 880) in MS patients and 158 pg/ml (125, 190) in non-MS controls (*p* < 0.001). The distribution was highly dispersed in the three clinical MS forms with no significant differences between them (*p* = 0.6) (Table 1; Figure 2A). The median CSF levels of CHI3L1 were 133 ng/ml (93, 215) in MS patients and 58 ng/ml (47, 77) in non-MS controls (*p* < 0.001), and differed between MS phenotypes being 119 ng/ml (81, 186) in RRMS, 139 ng/ml (95, 211) in SPMS, and 180 ng/ml (146, 265) in PPMS (*p* = 0.01) (Table 1; Figure 2A). Bonferroni *post-hoc* correction showed a significant difference between RRMS and PPMS (*p* = 0.009).

At the time of LP, 93 patients were considered active (78 RRMS, 9 SPMS, and 6 PPMS) according to the given definition of active disease (clinical attack and/or the presence of GEL in the MRI). CSF NFL levels in patients with and without active disease were 710 pg/ml (323, 1480), and 329 pg/ml (237, 614), respectively (*p* < 0.001; Figure 2B). This difference maintained statistical significance in RRMS (*p* = 0.016) and SPMS (*p* = 0.007) but not in PPMS (*p* = 0.265). Considering only the presence of GEL, NFL levels differed depending on whether GEL was present (740 pg/ml) (313, 1,719) or absent (385 pg/ml) (263, 710) (*p* < 0.001), but there was no evidence of a correlation with the number of this type of lesions (*p* = 0.395).

Median CSF CHI3L1 levels in active patients were 133 ng/ml (85, 217), which was not significantly different from patients without inflammatory activity [140 ng/ml (93, 220)] (*p* = 0.862; Figure 2). Irrespective of the MS phenotype was (*p* = 0.645), SPMS (*p* = 0.390), or PPMS (*p* = 0.267). CSF CHI3L1 levels in patients with or without GEL were not significantly different, being 133 ng/ml (87, 219) and 135 ng/ml (88, 196) (*p* = 0.705), and the number of GEL did not correlate with CHI3L1 levels (*p* = 0.395). Although, overall, median levels of CSF NFL, and CHI3L1 were high in MS, extreme values of both biomarkers were characteristic of RRMS patients with active disease (Figure 2B).

### Correlation of CSF NFL and CHI3L1 Levels With Other Clinical Variables

There was a significant correlation between CSF CHI3L1 and NFL concentrations (*r* = 0.46; *p* < 0.001; Figure 2D) but, when analyzing this correlation in distinct MS clinical forms it only persisted in RRMS (*r* = 0.58; *p* < 0.001) compared to SPMS (*r* = 0.15; *p* = 0.195), and PPMS (*r* = 0.3; *p* = 0.081). There was a trend between decreased CSF NFL levels and disease duration at the time of LP (*r* = −0.2; *p* = 0.02) or time from clinical relapse (*r* = −0.28; *p* = 0.04). NFL was higher in the presence of OCMB (652.0 pg/ml vs. 487.8 pg/ml; *p* = 0.02) only in the RRMS subgroup of patients (Figure 2D).

Higher concentrations of CSF CHI3L1 were associated with increased age (*r* = 0.2; *p* = 0.013) and higher EDSS at the time of LP (*r* = 0.21; *p* = 0.009), although correlations were weak. The presence of OCMB was more frequent with higher levels of CHI3L1 only in the RRMS subgroup (125 ng/ml vs. 108 ng/ml; *p* = 0.004), as it occurred with NFL (Figure 2C). OCMB was not associated to disease activity if we considered a clinical attack (χ^2^; *p* = 0.132) or presence of GEL (χ^2^; *p* = 0.519) separately, but patients with both conditions had OCMB in the CSF more frequently (χ^2^; *p* = 0.034). The mean number of GEL in the presence of OCMB was 2.53, while in its absence it was ere 1.36, but this observation did not reach statistical significance (*p* = 0.121).

At the time of LP, 109 patients were not treated, and 49 patients were treated (26 RRMS, 20 SPMS, 3 PPMS). Twenty-six patients were under first-line DMT (23 IFN-beta, 3 glatiramer acetate), 20 were on second-line DMT (2 fingolimod, 15 natalizumab, 3 antiCD20), and the remaining 3 had undergone ASCT in the previous 5 years after treatment failure. Median CSF NFL and CHI3L1 levels did not differ between patients being treated at time of LP (576 pg/ml and 135 ng/ml) and untreated patients (514 pg/ml and 123 ng/ml; *p* = 0.8 and *p* = 0.2, respectively).

### Predictive Value of CSF NFL and CHI3L1 in RRMS

After LP, our cohort was prospectively followed and clinical and disability data, including relapse rate, and date of conversion to progressive disease, were registered. Twenty-five RRMS patients (25%) experienced a 1-point increase in disability assessed with EDSS after LP. Bivariate Kaplan Meier analysis showed CHI3L1 (log rank *p* = 0.018) as potential predictor with a tendency for NFL (log rank *p* = 0.0523) and disease activity (log rank *p* = 0.109) to differ in 1-point EDSS worsening. After multivariable analysis, only CHI3L1 persisted as independent predictor {HR = 2.99 [95% CI (1.27, 7.07)]}. NFL had no impact on this outcome (Table 2).

**Table 2:** Cox regression analyses for 1-point EDSS worsening and diagnosis of progressive disease in RRMS patients during follow-up.

	**Estimate**	**Std. error**	**HR**	**Lower 95%**	**Upper 95%**	***p*-value**
**1-point EDSS worsening**						
log_10_(CHI3L1)	1.097	0.438	2.996	1.269	7.074	0.008
**Disease progression**						
log_10_ (CHI3L1)	2.893	1.050	18.044	2.305	141.3	0.0002
Disease activity	−1.682	0.607	0.186	0.057	0.611	0.002

*CHI3L1, chitinase-3-like-1*.

Fourteen RRMS patients (14%) progressed to SPMS. Bivariate Kaplan Meier analysis detected CHI3L1 (log rank *p* = 0.0001), NFL (log rank *p* = 0.015), and disease activity (log rank *p* = 0.001) as potential predictors for diagnosis of SPMS. With Cox analysis, only higher CHI3L1 levels, {HR = 18 [95% CI (2.31, 141.3)]}, and active disease {HR = 0.186 [95% CI (0.06, 0.62)]} were shown to be associated with disease progression after LP (Table 2). NFL was not independent predictor of disease progression.

Overall, escalation to second line DMT was needed in 57 patients during follow-up (9 fingolimod, 29 natalizumab, 14 rituximab, 2 ASCT, 1 alemtuzumab, 2 metotrexate). Bivariate Kaplan Meier analysis found gender (log rank *p* = 0.043), and EDSS (log rank *p* = 0.047) to be potential predictors for the need of treatment escalation. None had predictive value for this outcome after multivariate analysis.

Forty-two RRMS patients (42%) had a clinical relapse after LP. Bivariate Kaplan Meier analysis detected gender (log rank *p* = 0.043) and EDSS (log rank *p* = 0.046) as potential predictors for clinical relapse. None had predictive value for this outcome after multivariate analysis.

### Combined Assessment of CSF NFL and CHI3L1 Concentrations in Distinct MS Phenotypes

**Figure 3:**
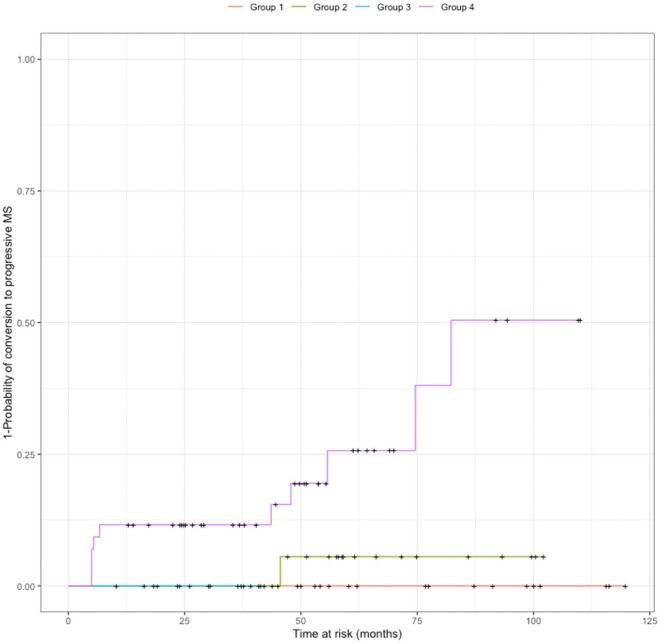
Probability of conversion to SPMS according to biomarker profile. Patients were cathegorized according to NFL and CHI3L1 median values (calculated in the RRMS cohort without disease activity) into four groups: Group 1: both NFL and CHI3L1 below median value; Group 2: high NFL; Group 3: High CHI3L1; Group 4: NFL and CHI3L1 above median values. Patients included in group 4 had more probability to be diagnosed of SPMS during follow-up than patients pertaining to the other groups (log rank *p* = 0.02). SPMS: secondary progressive multiple sclerosis.

With the purpose of investigating the value of the combined measure in MS prognosis, we stratified patients in four groups according to both NFL and CHI3L1 values with respect to their median in the RRMS cohort in the absence of disease activity (Group 1: both NFL and CHI3L1 below median value; Group 2: high NFL; Group 3: High CHI3L1; Group 4: NFL and CHI3L1 above median values). Bivariate analysis using biomarker profiles as predictors showed that being a patient in Group 4 was predictive of diagnosis of progressive disease during follow-up (log rank *p* = 0.02) compared to being included in any of the other groups of patients (Figure 3). Paired biomarker levels were not predictive of increasing 1-point in disability (log rank *p* = 0.094), need for escalation therapy (log rank *p* = 0.173) or relapse after LP (log rank *p* = 0.783).

**Figure 4:**
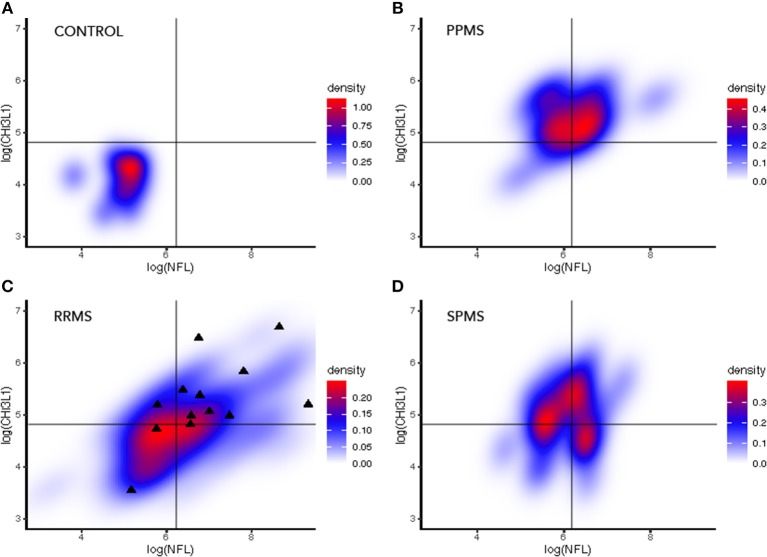
Density maps representing paired NFL and CHI3L1 values in CSF of non-MS controls and MS patients. The black lines in each diagram represent the median log values of CSF NFL and CHI3L1 in the whole cohort. Note that each subgroup has the peak density of patients in a different position in reference with the median log values. **(A)** In non-MS control group, almost all patients fell in the left-inferior quadrant. **(B)** In PPMS subgroup, CHI3L1 levels were over the median in almost all patients, and two groups were distinguished with regards to NFL levels. **(C)** In the RRMS group, black triangles represented patients that at the end of the observation period evolved to progressive disease. **(D)** SPMS patients constitute an intermediate density map with three regions of distribution. NFL, neurofilament light chain; CHI3L1, chitinase-3-like-1; RRMS, relapsing-remitting multiple sclerosis; SPMS, secondary progressive multiple sclerosis; PPMS, primary progressive multiple sclerosis; Switch, diagnosis of SPMS during follow-up.

The representation of paired values NFL/CHI3L1 of biomarkers (each pair corresponding to one patient), in density maps with a raster diagram, showed that peak density of patients was distinctly distributed between non-MS controls and MS patients, and within MS, between distinct MS clinical forms (Figure 4). RRMS patients that were diagnosed of SPMS during follow-up corresponded to patients with both NFL and CHI3L1 concentrations above median values.

**Figure 5:**
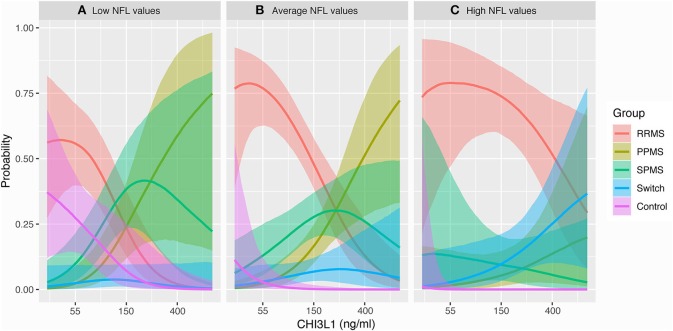
Marginal effect plots. This plot is the graphical representation of the multinomial regression model performed and shows the probability to be comprised in a MS clinical form, including the probability of conversion to SPMS, with regards to CSF NFL, and CHI3L1 levels. Thus, this diagram shows the interaction between both biomarkers. **(A)** When levels of NFL are low, low CHI3L1 levels imply higher probability to be a non-MS control (pink) or a RRMS patient (red). High CHI3L1 levels in these patients are associated with the diagnosis of progressive MS (green and brownish green). Probability of switching to SPMS is very low. **(B)** With average levels of NFL, the probability of pertaining to a non-MS control decreases, and CHI3L1 levels discriminate better between RRMS (low CHI3L1), and PPMS (high CHI3L1). **(C)** With high NFL levels, the probability of being comprised in the RRMS group is the highest. High NFL and CHI3L1 are more probable in patients diagnosed of SPMS during follow-up, and in a subgroup of PPMS patients, likely PPMS patients with active disease. NFL, neurofilament light chain; CHI3L1, chitinase-3-like-1; RRMS, relapsing-remitting multiple sclerosis; SPMS, secondary progressive multiple sclerosis; PPMS, primary progressive multiple sclerosis; Switch, diagnosis of SPMS during follow-up.

We additionally tested the interaction between both biomarkers to discriminate MS phenotypes and predict progression using a multinomial logistic regression model (Table 3). The analysis showed a discriminating value of individual measures of CSF NFL and CHI3L1 between RRMS and SPMS [HR 0.41 (0.18–0.82)] and between RRMS and PPMS [HR 0.46 (0.19–0.87)]. An interaction between NFL and CHI3L1 in the multinomial regression model did not show statistical power to predict diagnosis of SPMS {0.96 [95% CI (0.54, 1.63)]}. However, the representation of these data in a marginal effects plot showed that the probability of switching to progressive disease was characteristic of patients with higher concentrations of CSF NFL and CHI3L1 (Figure 5C). MS phenotypes corresponding to low and median NFL levels are depicted in Figures 5A,B.

**Table 3:** Multinomial regression model for combined NFL-CHI3L1value in clinical stage discrimination.

	**Estimate**	**Std. error**	**Exp (Estimate)**	**Lower 95%**	**Upper 95%**
PPMS Intercept^a^	−1.552	0.34	0.212	0.102	0.394
SPMS Intercept	−0.819	0.239	0.441	0.267	0.697
Switch Intercept	−2.198	0.376	0.111	0.049	0.219
Non-MS controls Intercept	−7.931	2.44	0	0	0.018
PPMS logCHI3L1	**1.644**	0.388	**5.173**	**2.514**	**11.199**
PPMS logNFL	–**0.983**	0.446	**0.374**	**0.149**	**0.862**
PPMS logCHI3L1: logNFL	–**0.771**	0.437	**0.462**	**0.186**	**0.975**
SPMS logCHI3L1	**0.804**	0.311	**2.235**	**1.233**	**4.181**
SPMS logNFL	–**0.948**	0.34	**0.387**	**0.195**	**0.723**
SPMS logCHI3L1: logNFL	–**0.896**	0.399	**0.408**	**0.177**	**0.819**
Switch logCHI3L1	**0.924**	0.445	**2.518**	**1.098**	**6.293**
Switch logNFL	−0.064	0.419	0.938	0.415	2.129
Switch logCHI3L1: logNFL	−0.04	0.285	0.961	0.544	1.631

a *The reference category is RRMS. RRMS, relapsing-remitting multiple sclerosis; SPMS, secondary progressive multiple sclerosis; PPMS, primary progressive multiple sclerosis; Switch, diagnosis of SPMS during follow-up. logNFL, log-transformed neurofilament light chain levels; logCHI3L1, log-transformed chitinase-3-like-1 levels. Significant results are highlighted with bold letters*.

## Discussion

This study shows that MS clinical phenotypes and disease activity are associated with CSF NFL-CHI3L1 levels when assessed both individually and combined. NFL levels alone seemed to be more indicative of disease activity, whereas CHI3L1 levels were related to progression of disability as previously shown. As a novelty, here we state that both biomarkers in combination could be helpful to classify MS patients into distinct clinical forms, and to predict diagnosis of SPMS in the RRMS cohort.

We confirmed previous reports in which NFL levels were higher in MS patients compared to non-MS-controls, and further increased when clinical attack and/or GEL occurred (37). As time from relapse passed, CSF NFL levels decreased to median values, reinforcing the relationship between the NFL peak level, and acute axonal damage. We did not find a correlation of NFL with age, as it had been previously reported (8), probably because there were two opposing phenomena occurring in parallel: the disease activity being higher in younger individuals (38) and the age-associated axonal degeneration or neurological co-morbidities associated with older individuals (39). This is why we did not use age-stratified reference values extracted from a non-MS cohort. Instead, we used median values from the RRMS cohort without disease activity as the threshold, and ANCOVA analysis was performed to adjust all our comparisons by age, gender, and disease duration.

CSF CHI3L1 levels were also high in MS compared to non-MS controls as previously reported (19, 40). Recent studies showed that CSF CHI3L1 levels were increased in progressive MS (24). Although median values of CSF CHI3L1 in MS were not related to disease activity, an observation also reported for plasma levels (24), some RRMS patients with active disease and particularly elevated CSF NFL concentration also presented increased CSF CHI3L1 levels (even 2–3 times the median values). To explore whether this increase was related to the disruption of the cerebrospinal fluid barrier during acute inflammation, the presence, and the number of GEL were both assessed, and correlation analyses were performed. We did not find an association between CSF CHI3L1 levels and presence of GEL, nor increasing numbers of GEL. This might imply that during a clinical relapse, other immunologic pathways than those causing GEL are responsible of CHI3L1 secretion. In fact, it has been demonstrated that an IL-13 pathway activation is related to high CHI3L1 levels (41) with differential patterns in bacterial or viral meningitis, suggesting the involvement of CHI3L1 in heterogeneous inflammatory pathways.

CSF CHI3L1 is also very dependent on age (40). Patients with progressive disease are usually older than RRMS patients, and therefore, the increase in CSF CHI3L1 levels could be merely explained by the abnormal priming of CNS glia that occurs in older ages [reviewed in (42)]. Although correlation of CHI3L1 with age was weak in our study, we adjusted all the analyses by age and disease duration to overcome the potential effect of age-dependent glial priming in our results.

OCMB is a known CSF biomarker of inflammatory disease and implies a worse prognosis in MS (31). In our cohort, its presence correlated with CSF NFL and CHI3L1 levels in RRMS patients, but we did not find an association with these biomarkers in progressive MS. This, and the fact that we found OCMB more frequently in patients with both clinical attack and GEL, suggests the more prominent role of this biomarker in acute inflammation than in chronic inflammation. OCMB positivity in our cohort was not able to predict disability accrual, diagnosis of progressive disease, occurrence of relapse, or treatment escalation.

Regarding the value of both CSF biomarkers in prognosis, recent reports in a CIS/RRMS cohort did not find a prognostic influence for these biomarkers in progression (43). Although we found that CSF NFL was predictive of disability accrual and prospective diagnosis of progressive disease in the bivariate analysis, it was not selected by the multivariable analysis as an independent predictor. In contrast, CHI3L1 alone was predictive of 1-point EDSS worsening and of the re-assignation of patients to progressive MS during follow-up. The median time for conversion to SPMS is normally >10 years as reported in natural history studies (44), and therefore, it is important to remark that our cohort was composed of MS patients with distinct clinical forms and at different time points from disease onset (with disease duration times up to 19 years), while the cohort studied by Sellebjerg et al. included only patients with recent diagnosis and who were followed for a median time of 5.7 years.

Further, we analyzed the usefulness of the combined measure of CSF NFL and CHI3L1 levels. Both values provided supplementary information of the MS phenotype with higher values of NFL that seemed to correspond more frequently to active patients (most in the RRMS subgroup) susceptible to respond to current DMT, while higher values of CHI3L1 were associated with progressive MS (more evident for PPMS). Eleven over a total of 14 RRMS patients diagnosed of SPMS during follow-up had high levels of both NFL and CHI3L1. This led us to investigate the combination of biomarkers and the ability to predict the diagnosis of progressive MS in patients from the RRMS cohort. We found that having high levels of CSF NFL and CHI3L1 was predictive of switching to the SPMS group. However, the number of patients that progressed during the study was low and CSF was collected at distinct time points from disease onset, hence we cannot infer from our data whether biomarkers could inform likeliness of conversion or were, indeed, detecting subclinical progression. Supporting the latter was the assessment of longer disease duration in these patients.

A disadvantage of measuring biomarkers in the CSF is the invasiveness nature of LP. This prevents for the use of CSF NFL and CHI3L1 levels in longitudinal studies. Currently, new detection methods allow precise detection of NFL in serum, and therefore allow for multiple measures during follow-up (12). Nevertheless, serum CHI3L1 levels are not specific of MS (45). In this report, we show that prognostic information can be obtained from a diagnostic lumbar puncture at any time from disease onset. Given the retrospective nature of the study, results should be interpreted cautiously, and our conclusions need to be confirmed by prospective analysis of larger cohorts. However, we would like to emphasize, as a strength of the study, that all patients had simultaneous assessment in CSF of NFL and CHI3L1 levels, OCMB, together with MRI and disability data, with a median follow-up of 50 months. The relationship of these biomarkers with OCMB and the value of the combined measure for MS profiling and recognition of progressive disease, as far as we know, had not yet been reported.

To conclude, individual measures of CSF NFL and CHI3L1 are biomarkers of disease activity and progression, respectively. The pattern of combined measure discriminates MS phenotypes and predicts the subset of RRMS patients that will progress clinically allowing early intervention. Whether early intervention guided by biomarkers can change the natural history of the disease is unknown, and should be the focus of future studies.

## Data Availability

The datasets generated for this study are available on request to the corresponding author.

## Ethics Statement

All patients provided written informed consent. The study was approved by the Institutional Ethics Committee in Hospital Universitari I Politècnic La Fe (reference number PI17/01544).

## Author Contributions

SG-P and BC have designed the study, analyzed data, and wrote the manuscript. SG-P additionally carried out the data collection and statistical analysis. LC-N and JC-V have processed CSF and performed ELISA. BC has been attending physician and designed the database, the treatment protocol, and had been responsible with FP-M, FG, CA, and JD for the clinical follow-up. JG-M helped in collecting data. RG helped in designing figures and revising the manuscript. DH supervised the statistical analysis and helped in figure design.

### Conflict of Interest Statement

The authors declare that the research was conducted in the absence of any commercial or financial relationships that could be construed as a potential conflict of interest.
